# Highly distinguishable isomeric states of a tripodal arylazopyrazole derivative on graphite through electron/hole-induced switching at ambient conditions

**DOI:** 10.3762/bjoc.21.112

**Published:** 2025-07-22

**Authors:** Himani Malik, Sudha Devi, Debapriya Gupta, Ankit Kumar Gaur, Sugumar Venkataramani, Thiruvancheril G Gopakumar

**Affiliations:** 1 Department of Chemistry, Indian Institute of Technology Kanpur, Kanpur 208016, UP, India; 2 Department of Chemical Sciences, Indian Institute of Science Education and Research (IISER) Mohali, Sector 81, SAS Nagar, Knowledge City, Manauli, Punjab, India

**Keywords:** azo compounds, electron/hole-induced isomerization, isomerization, molecular switches, switching

## Abstract

Manipulating the energy barrier and extending the half-life of nonequilibrium states in photochromic switches presents viable solutions for applying them in molecular electronics. Typically, the half-life of the *Z* isomer of azobenzene (AB) is a few days. Arylazopyrazole-based molecular switches are one of the profoundly explored systems in recent times due to their superior bidirectional photoswitching and long half-life (over a thousand days at room temperature) of *Z* isomers. Herein, we utilize an efficient solid-state photoswitchable fluorinated tripodal *N*-functionalized arylazo-3,5-dimethylpyrazole derivative (FNAAP) to envisage and access multiple metastable states on the surface. The tripodal molecule forms well-ordered, large crystalline domains on graphite through non-bonding interactions between the molecules. By injecting electron/hole pairs into the self-assembled molecules on a surface using a scanning tunneling microscope (STM) tip, they are switched between 8 states (*EEE*, *EEZ*, *EZE*, *ZEE*, *EZZ*, *ZEZ*, *ZZE* and *ZZZ*) in a tunneling junction at ambient conditions. Contrary to the degeneracy-controlled four states in solution phase, all the eight states are remarkably stable on the surface and are well distinguishable by the tunneling current passing through the molecule at the tunneling junction. The change in current upon switching between these states is approximately an order of magnitude. This is particularly notable at positive sample voltage compared to negative sample voltage. The exceptional stability of the states at ambient conditions provides an opportunity to use a single FNAAP molecule as an 8-bit operation unit, with a potential storage capacity of ≈200 Tbits per 1 cm^2^ area using an atomically precise write and read tool like an STM tip.

## Introduction

Molecular electronic devices have been predicted to outperform silicon-based devices [1–2]. Reprogrammable arrays of molecular switches with multiple states [3], logic-in-memory operations using single-metallofullerenes [4], multistate molecular switches [5], molecular memory and logic processing based on guest capture/release [6], etc., have demonstrated the possibility of using single molecules in logic operations. Manipulating molecular spin states [7–9] and electronic states [10–11] using tunneling electrons/holes have shown the potential to revolutionize quantum information processing. For a single molecular switch possessing multiple spins [7–9] or electronic states [10–11], the functional unit can be suitably triggered through external stimuli to induce the switching and to modulate the function. Photo-triggerable molecular switches like azobenzene (AB) [10–13], spiropyran [14], diarylethene [15] and multifunctional AB [16] have attracted a lot of interest due to the distinct differences in the electronic and optical properties of switchable states.

The applicability of molecular switches relies on the efficiency, reversibility, and the half-life associated with different non-equilibrium states. Typically, the half-life of the *Z* isomer of AB is a few days [17]. Chemical modifications of AB derivatives play a crucial role in realizing efficient bidirectional photoswitches with longer half-lives [18–19]. In particular, replacing one of the phenyl units in AB with a five-membered heterocycle and the resulting *Z* isomer of phenylazoheteroarenes has extended half-life ranging up to 1000 days and imparted quantitative and reversible photoswitching [20–21]. The long-term photoswitching stability and tunable half-lifes of *Z* isomers of azopyrazole-based switches [20–22] have made their way to several applications in thermal energy storage [23], photoswitchable inhibitor [24], photoswitchable hydrogelator [25], photoregulation of DNA nanosystems [26], in controlling surface wettability [27], and in solar energy conversion [28]. Considering these advantages, we synthesized *C*_3_ symmetric tripodal azopyrazole-based derivatives having a trimesoyl core and aroylazole connections as a solid-state switchable probe for light-driven reversible printing and erasing applications [29]. Besides exhibiting excellent photoswitching in solution phase, the tripodal photoswitches exhibit extended half-life of the *ZZZ* isomer in the solid-state as well [22,29]. The overall efficiency of the molecular switch could be attributed to the electronically decoupled individual photoswitchable units by 1,3,5-functionalization and attainment of non-planar geometry [30]. Molecular switches with multiple switching units having extended lifetime for their non-equilibrium states will be a unique tool for fabricating multi-functional molecular switches on the surface. Molecular switches with multiple switching units are scarce on the surface [10,31] and mostly studied under low temperatures. If such multi-functional molecular switches, with stable non-equilibrium states, can be reversibly switched under ambient conditions, quantum information processing based on single molecules can be revolutionized.

**Figure 1:**
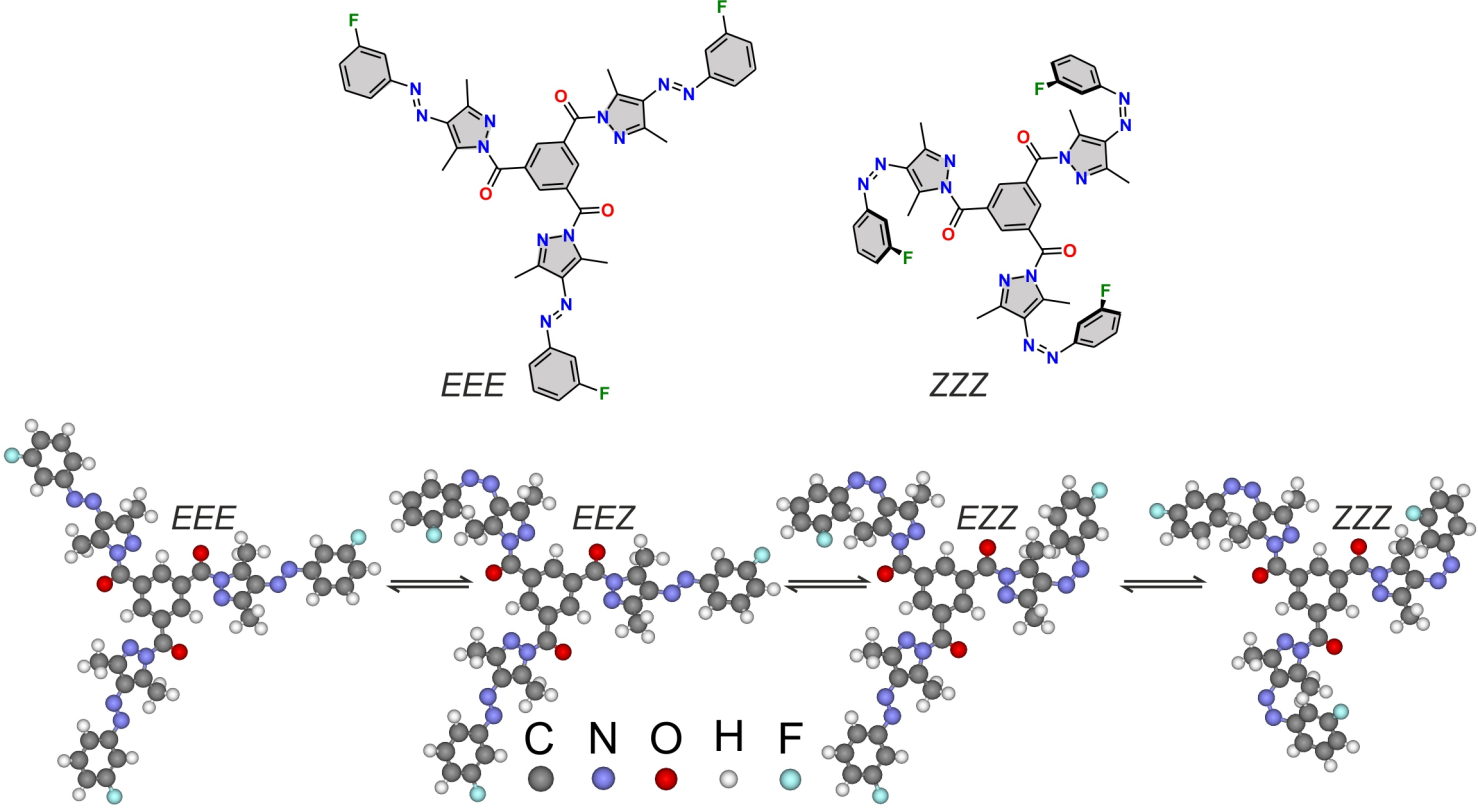
Top panel: Chemical structures of *EEE*, and *ZZZ* isomers of (FNAAP). Lower panel: Geometry-optimized ball and stick model of FNAAP. The *EEE*, *EEZ*, *EZZ* and *ZZZ* isomers are shown in sequence from left to right, respectively.

In this article, we show the single molecule electron/hole-induced switching of a fluorinated tripodal *N*-functionalized arylazo-3,5-dimethylpyrazole derivative (FNAAP), a multi-functional molecular switch, on a graphite surface at ambient conditions. The fluorine substitution is chosen in FNAAP for its better photoisomerization efficiency (in solution as well as in solid state) and higher molecular stability [29]. Possible photoisomers of FNAAP, *EEE*, *EEZ*, *EZZ* and *ZZZ* are shown in Figure 1. FNAAP molecules assemble into well-ordered large domains on graphite and are revealed by atomic force microscopy (AFM) and scanning tunneling microscopy (STM) studies. The molecular level arrangement in the assembly reveals a hexagonal packing through weak non-bonding interactions between the carbonyl and aromatic hydrogen, and is further understood by reactive force-field (ReaxFF) simulation [32]. Using electron/hole under a tunneling junction, molecules are switched between 8 stable states (*EEE*, *EEZ*, *EZE*, *ZEE*, *EZZ*, *ZEZ*, *ZZE* and *ZZZ*) at ambient conditions. These states are remarkably stable and are well distinguishable by the tunneling current passing through the molecule at the tunneling junction. The electron/hole-induced switching reveals that the electron injection yields a higher switching probability than that through hole injection. It is also observed that the overall switching probability of FNAAP on the surface is several-fold higher than that of molecular switches with a single switching unit known. The comparison is made for an AB derivative (4-(phenylazo)benzoic acid) (PABA) and a pyrazole-AB derivative (*E*-4-((1,3,5-trimethyl-1*H*-pyrazol-4-yl)diazenyl)benzoic acid) (PyABA) on a graphite surface under similar experimental conditions. The choice of molecules that can be studied under ultra-high vacuum (UHV) is limited due to the requirement of their thermal stability and UHV compatible sublimation temperature. Therefore, the solution-based preparation method and solid-state measurements at ambient conditions hold their importance in exploring novel and thermally less stable molecular switches.

## Results and Discussion

**Figure 2:**
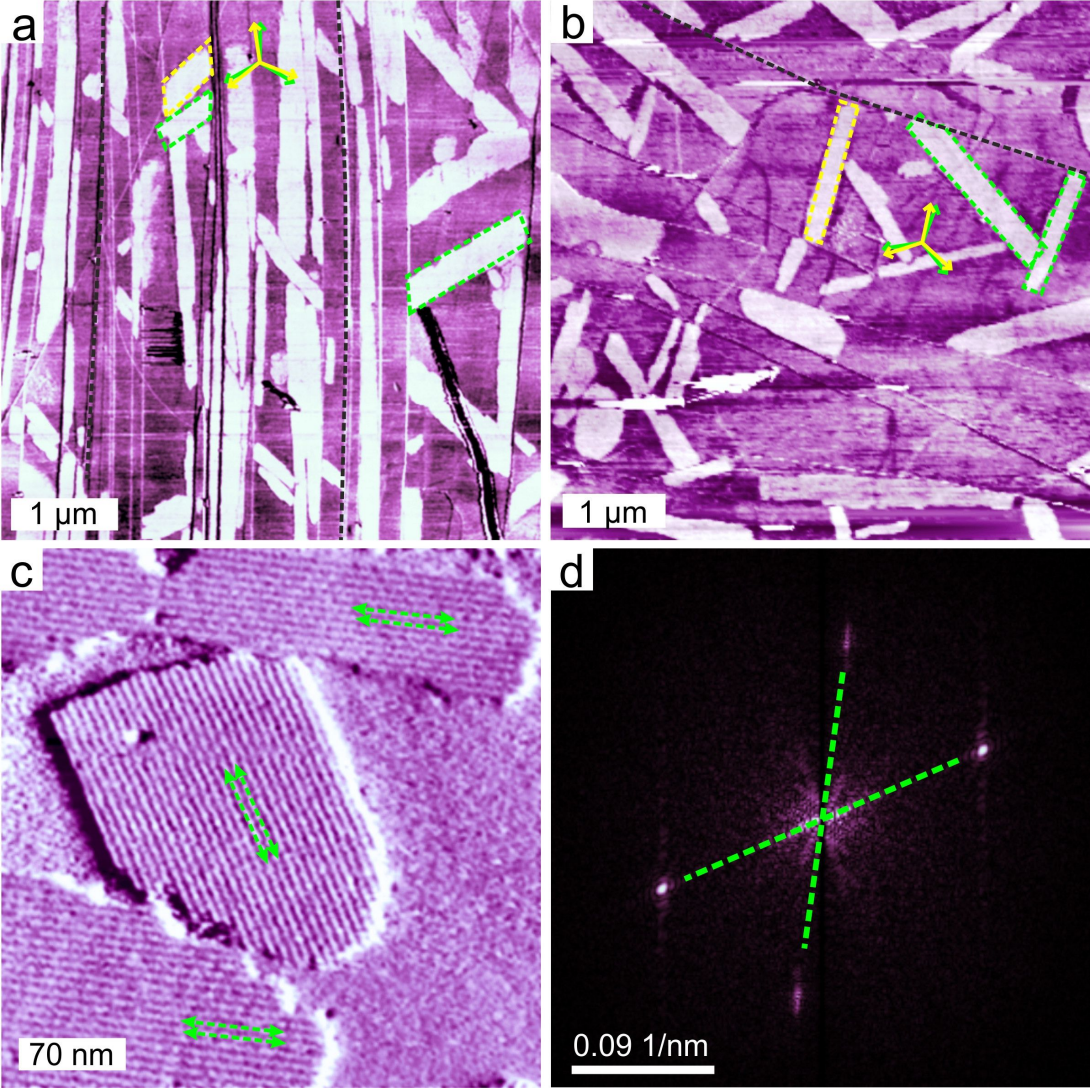
AFM phase images (a, b and c) of ultra-thin films of FNAAP deposited from ethanolic solution on HOPG (0001) and are obtained from independent areas. Domains in the 1D phase of FNAAP are marked by green and yellow dashed lines. The green and yellow three-fold arrows depict the orientation of the long edges of the islands with respect to each other. A few graphite step edges are marked by black dashed lines. Double-headed arrows in (c) indicate line-like features within the domains of the 1D phase, which is due to the moiré pattern originating from the super-lattice. (d) 2D-FFT taken on (c). The resonances corresponding to the super-lattice are indicated by green dashed lines. Two resonances are rotated by ≈120° and correspond to the major orientations of the domains in the 1D phase.

Typical AFM phase images of an ultra-thin film of FNAAP deposited from ethanolic solution on highly oriented pyrolytic graphite (HOPG) are shown in Figure 2. The corresponding topographs are shown in Supporting Information File 1. The AFM images of the ultra-thin film reveal domains of assembled molecules shown as bright contrast, marked by green and yellow dashed lines in Figure 2a,b. The pristine graphite surface has a median contrast. The molecular domains are growing as long islands with well-defined growth facets. We tag the domains as one-dimensional (1D) phase, as noted by their 1D growth. The typical apparent height of the FNAAP islands is ≈0.7 nm, indicating that the islands are monolayers (see Supporting Information File 1, section 2 for height profile of islands). The apparent height measured is consistent with monolayer islands of planar molecules [33]. It is also noted that the molecular islands are typically oriented by ≈0°, 120°, and 240°, with respect to the long edges of the islands. The angles of orientation are determined using a 2D fast Fourier transform (2D FFT) of high-resolution images, and an example is shown in Figure 2d. The resonances in the 2D-FFT are originated from the moiré pattern of the super-lattice. Two distinct orientations are visible in the 2D-FFT. The 3-fold orientation of islands suggests that the molecular lattices are oriented with respect to the graphite lattice, which has 3-fold symmetry [33–35]. Each of the orientations has a mirror domain as well, making a total of six orientations for the islands on the surface. The orientations of the islands are marked by yellow and green 3-fold arrowheads in Figure 2a,b. The mirror orientations are originating due to the incommensurate alignment of the molecular lattices along the graphite compact lattices at an angle. The angle between the mirror domains is ≈7°, that is, the molecular lattice is oriented by ≈3.5° with respect to the graphite compact lattice. This clearly suggests that the molecular lattice is not a commensurate lattice with respect to graphite [33–35].

A high-resolution AFM phase image of a few islands in 1D phase is shown in Figure 2c. Molecular domains reveal line-like features within the domains and extend through the length of the islands. Adjacent line-like features are indicated by green double-headed arrows and are separated by 6.3 ± 0.1 nm. The magnitude of the spacing suggests that the line-like features are originating due to a super-periodic pattern of molecular assembly, namely a moiré pattern [33–35]. This further suggests that the molecular adlayer is forming an incommensurate lattice with respect to the graphite lattice and leading to a super-lattice.

In addition to the 1D phase, we also observed a two-dimensional (2D) phase, named due to the 2D nature of the growth. We noted that the surface is covered by ≈88% of the 1D phase, and the remaining is covered by the 2D phase. Interestingly, high-resolution images revealed no line-like features due to the moiré pattern within the islands in the 2D phase. As suggested earlier, the moiré pattern is typical for incommensurate structures. Thus, we propose that in the 2D phase, the molecules organize commensurate with respect to the graphite lattice. AN AFM image corresponding to a 2D phase of FNAAP is provided in Supporting Information File 1, section 3. It is to be noted that the majority of the islands in the 2D phase appear with nearly hexagonal-like facets, indicating that they are most likely originating from a hexagonal type of lattice.

**Figure 3:**
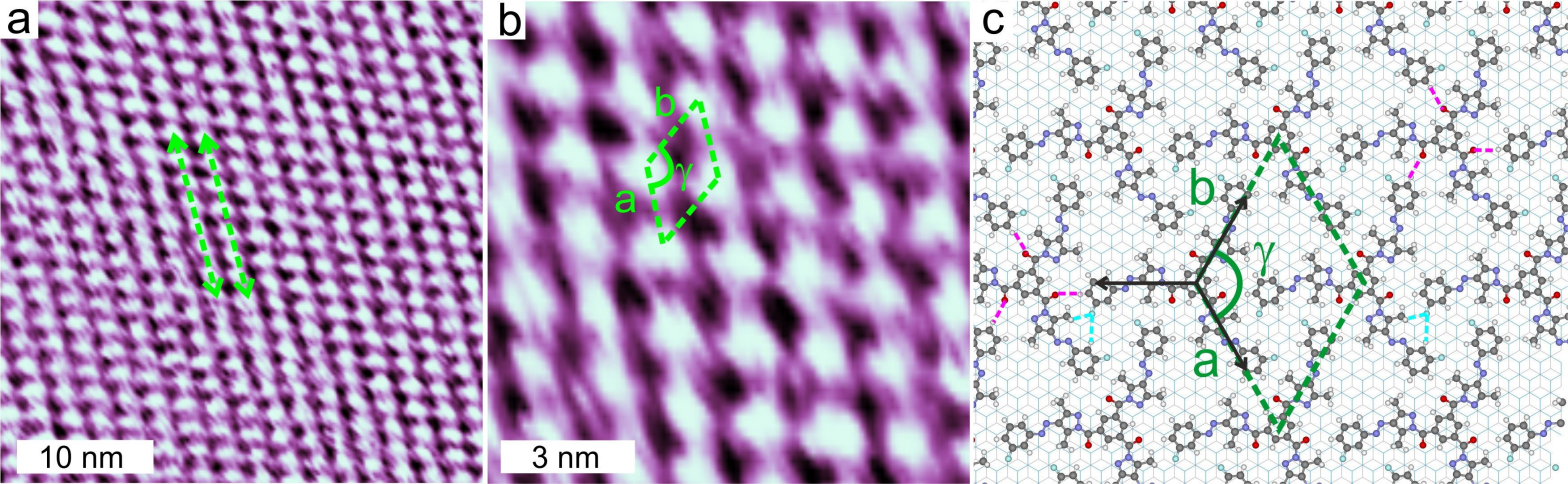
Constant current STM topographs (300 pA, 0.3 V) of the FNAAP adlayer on HOPG (a, b) deposited from the corresponding ethanolic solution. Imaging is performed at the HOPG–air interface. Green dashed lines depict two adjacent molecular lattices, and the green dashed oblique depicts the unit cell of the assembly. (c) Force field optimized geometry of the adlayer of FNAAP (*EEE* isomer) on bilayer graphite. The unit lattice vectors of the adlayer are *a*, *b* and γ is the angle between the vectors. Black arrows indicate the compact lattice directions of graphite. Magenta dashed lines depict possible non-bonding interactions between carbonyl groups and aromatic hydrogen atoms. Blue dashed lines depict the same between fluorine and aromatic/methyl hydrogen atoms.

To further understand the microscopic structure of the assembly, we have performed STM experiments on ultra-thin films of FNAAP. Figure 3a,b shows constant current STM topographs of ultra-thin films of an FNAAP adlayer on HOPG (0001) at different resolutions. Single molecules appear as bright spots, and the arrangement of the molecules reveals a hexagonal packing. The green dashed arrow indicates two adjacent molecular lattices, and the green oblique indicates the unit cell of the assembly. The unit lattice vectors are *a* (1.9 ± 0.1 nm), *b* (1.9 ± 0.1 nm) and γ (≈120°), the angle between the vectors. The intermolecular distance obtained from STM corresponds to the lateral length of the *EEE* isomer (F to F distance is 22.06 Å). Thus, we conclude that the adlayer is formed by the energetically most favorable *EEE* isomer. This is also consistent with the NMR data, which reveals that the majority (>98%) of molecules in the solution are in the thermodynamically stable *EEE* form (see Supporting Information File 1, section 4 for a UV–vis spectrum of FNAAP in ethanol–chloroform mixture, which resembles the reported spectrum in chloroform [29]). Long 1D islands are visible in large-area STM images as well. A super-periodic pattern is visible at low resolution, and the periodicity (≈6.1 nm) matches with the AFM measurements. Additional STM images of the ultra-thin film of FNAAP at different resolutions are provided in Supporting Information File 1, section 5.

Next, we propose a molecular-level model for the adlayer of the *EEE* isomer of FNAAP. Based on the experimental lattice parameters, an initial geometry was modelled and subjected to geometry optimization using ReaxFF. The optimized assembly of FNAAP on bilayer graphite is shown in Figure 3c. The calculations assume a commensurate hexagonal lattice. The graphite lattice directions are marked by the 3-black arrows. The unit cell parameters of optimized geometry (*a* = 1.968 nm, *b* = 1.968 nm and γ = 120°) match well with the experiments. The microscopic arrangement of the molecules is clearly visible. It is to be noted that the fluorine atoms are oriented toward the aromatic and aliphatic hydrogen and the carbonyl group to aromatic hydrogen. This suggests possible directional non-bonding interactions between the molecules. For molecules with 3-fold symmetry, the 3-fold orientation within the assembly is not a surprise [10,36]. Therefore, it is summarized that the typical intermolecular interactions are of van der Waals type and interactions dominantly driven by steric packing. We note that the STM experiments reveal only a hexagonal type of arrangement of molecules, as shown in Figure 3 (additional STM images are provided in Supporting Information File 1, section 5). Based on the microscopic details obtained from STM and AFM images, we propose possible geometric models for incommensurate lattice and commensurate lattice and are included in Supporting Information File 1, section 6. Both incommensurate (*a* = 1.96 nm, *b* = 1.86 nm and γ = 117.6°) and commensurate lattices (*a* = 1.97 nm, *b* = 1.97 nm and γ = 120°) are hexagonal-type with slightly different lattice parameters. Thus, we summarize that both 2D and 1D phases have comparable arrangements of molecules with slightly different lattice parameters. This is also possibly the reason why we observe only a hexagonal-type of lattice in the STM experiments. Since the incommensurate lattice leads to the formation of super-periodicity, we observe a moiré pattern (line-like pattern) for the 1D phase (see Supporting Information File 1, section 6 for details).

**Figure 4:**
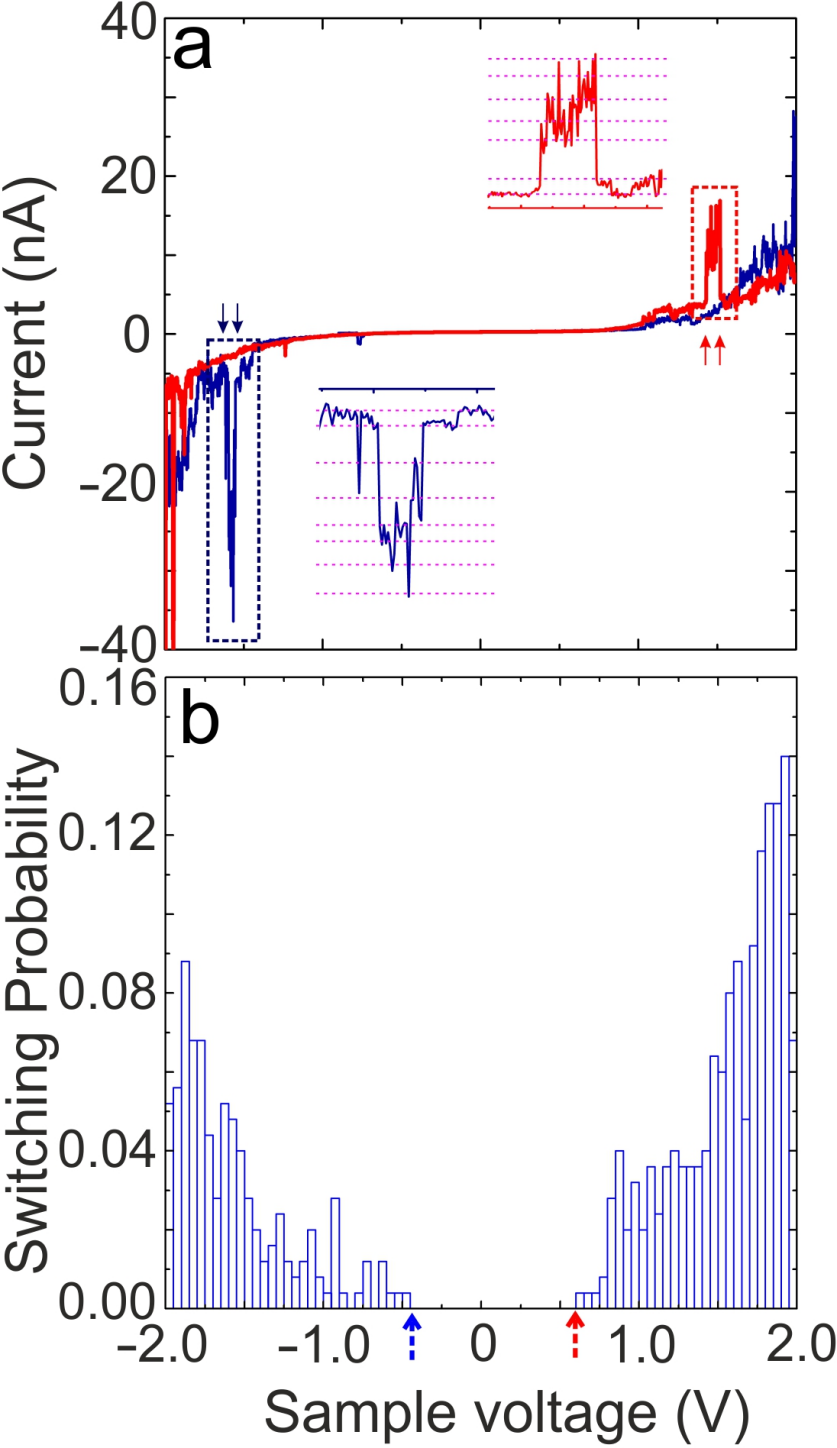
(a) Current versus sample voltage (*I*–*V*) recorded on a single FNAAP within the assembly. The *I*–*V* measurements are recorded by stabilizing the current at ≈0.5 nA and at −2 V. Arrows indicate the switching of FNAAP molecules between *E* and *Z* forms. Insets show the zoomed section of the *I*–*V* curves indicated using dashed rectangles. Distinct levels of current can be discerned and are indicated by dashed magenta lines. (b) Statistical analysis of all the switching events (voltage value corresponding to the up and down arrows). The switching probability is obtained by taking the ratio of the number of times switching is observed in a given voltage window to the total number of *I*–*V* measurements performed. The binning of *I*–*V* measurements is 10 mV. Each data point depicts the average probability for switching in intervals of 50 mV. Blue and red arrowheads depict the threshold voltage for switching at negative and positive sample voltages, respectively.

**Figure 5:**
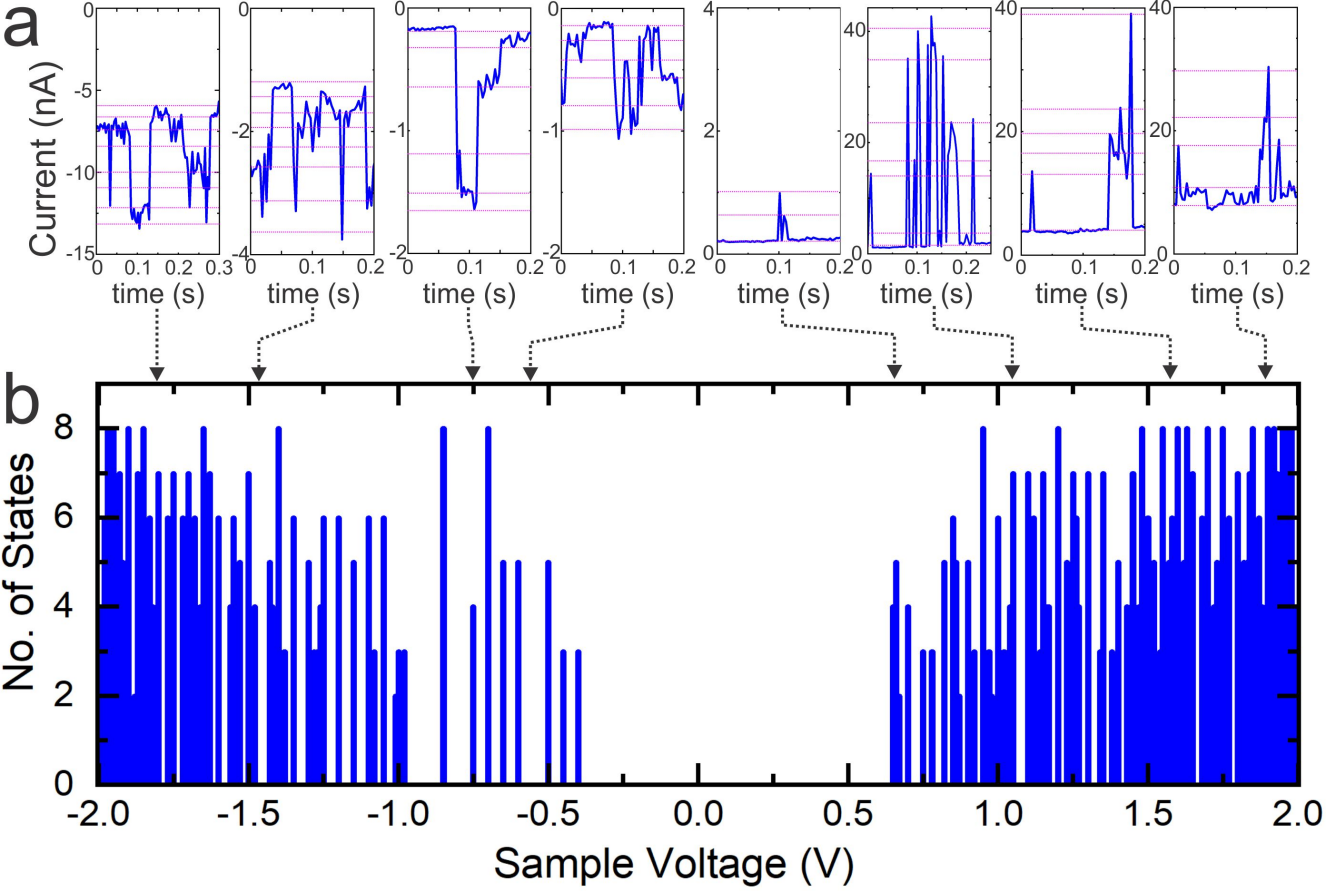
(a) Current versus time (time trace) at selected voltage intervals acquired on the adlayer of FNAAP on HOPG at ambient conditions. The time traces are part of *I*–*V* curves measured by stabilizing the feedback at ≈0.5 nA and at −300 mV. The mean value of the sample voltage at which the time traces are recorded is marked by dashed arrows. Magenta dashed lines represent stable recurring levels of current, which correspond to different isomeric states. (b) Statistical analysis of the number of states as obtained from multiple time traces in intervals of ≈100 mV voltage versus sample voltage.

Next, we show the electron/hole-induced *E*–*Z* isomerization of a single FNAAP within the assembly. By placing the STM tip on selected molecules, the sample voltage is ramped, and the corresponding change in current is measured. These measurements are tagged as current–voltage (*I*–*V*) characteristics. Electrons and holes are injected into molecules at negative and positive sample voltages, respectively. It is known that upon injection of electrons or holes, molecules get transiently charged on the graphite surface and undergo isomerization (switch) [11,37–38]. Figure 4a shows two independent *I*–*V* measurements obtained on two single FNAAP molecules. Additional *I*–*V* measurements are provided in Supporting Information File 1, section 7 for reference. Unlike the expected continuous variation of current as a function of voltage, we observed abrupt increases and decreases in the tunneling current, as indicated using up/down arrow heads. The abrupt change in current is an indication of geometrical changes at the tip-molecule tunneling junction. We presume that the change in current is due to the switching of molecules and is in accordance with previous reports [7,10–12,15,33,38]. Insets of Figure 4a show a zoomed section of the *I*–*V* curves indicated using dashed rectangles. Distinct levels of current can be discerned and are indicated by dashed magenta lines. We attribute these fluctuations in the current to the interconversion between different possible geometrical isomeric states of the molecule, that is, reversible *E–Z* isomerization. It will be shown later that the isomerization is electron/hole-induced, which is initiated by injecting electrons into molecular orbitals [7,10–12,15,33,38]. It is to be noted that within a given interval of voltage/time, the current repeatedly goes through similar values. The fluctuations in current have a step-like nature, and they can be clearly established by following the dashed magenta lines. Thus, it is concluded that the origin of the fluctuations in current is due to distinct reversible isomeric changes of the molecule; i.e., molecules go through different geometrical isomerization multiple times. These distinct levels and fluctuations in the current further confirm that they are not random noise or instabilities but switching between geometrical isomeric states of FNAAP. If the fluctuation in current was simply due to environmental instabilities, we would not have obtained *I*–*V* curves with such reproducible fluctuation (step-like nature) in the current. Additionally, the well-defined step-like nature of the fluctuation in current is visible in the time traces given in the upper panel of Figure 5. It is interesting to note that the abrupt changes are reversible (each increase in current is associated with a step involving a decrease in current), which indicates the molecular switching between two associated states.

The geometrical isomerization between the *EEE* and *ZZZ* forms of the molecule leads to structural changes, and the average distance between the tip and molecules is expected to change. The current changes drastically as it is exponentially dependent on the distance. Once a molecule is injected with an electron/hole, it changes its geometry, which in turn, causes a decrease or increase in the tunneling gap and thus the current changes abruptly. In other words, the abrupt change in current is caused by isomerization or geometrical changes in the molecule. The change in tunneling current has been earlier used to identify geometrical changes/isomerization of spin-cross-over molecule [7] and azobenzene derivatives [10–12,15,33,38] on the surface. Other degrees of freedom, like molecular vibrations, are also associated with configurational switching. However, such small changes may not lead to a distinguishable signal (change in current) in the *I*–*V* at ambient conditions. Therefore, we mainly rely on the sharp and abrupt change in current that is highly distinguishable from the background noise.

To understand the switching further, we have performed a statistical analysis of the voltages associated with every switching event (indicated by up or down arrows). Figure 4b shows the statistical analysis of all the switching events observed. We have included *I*–*V* curves recorded on 160 independent locations (each presumably from a few molecules) from different molecular domains. Some of the *I*–*V* curves used for the analysis are shown in Supporting Information File 1, section 7. The number of switching events is normalized by the number of *I*–*V* measurements used for the analysis. Thus, the ratio of the number of switching events to the number of *I*–*V* used directly corresponds to the switching probability. The following observations are noted from the statistical analysis. 1) a threshold voltage is observed for the isomerization of the molecules at both positive and negative sample voltages (marked by red/blue arrow heads); 2) the switching probability increases exponentially with increasing voltage; 3) the switching probability at positive voltage is almost double than that at negative sample voltage; 4) a strong asymmetry in the switching probability induced by electrons and holes.

The threshold voltage of switching suggests that the switching is electron/hole-induced [7,10–12,15,33,38]. The threshold voltage observed for the electron injection (marked by red arrowhead) and hole injection (marked by blue arrowhead) should match with the position of lowest unoccupied molecular orbital (LUMO) and highest occupied molecular orbital (HOMO), respectively for electron/hole-induced switching [7,10–12,15,33,38]. To understand the same, we have calculated the molecular orbitals at the B3LYP/6-311g level of theory using Gaussian 03. The computed HOMO–LUMO gap of *EEE*, *EEZ*, *EZZ* and *ZZZ* isomers is 3.59 eV, 3.27 eV, 3.26 eV, and 3.35 eV, respectively (see details in Supporting Information File 1, section 8). The HOMO–LUMO gap obtained from the calculation is much higher than the gap obtained from the threshold voltage, which is ≈1.1 V. This suggests that while switching, the molecules may not be in their neutral state. Since the graphite–molecule interaction is weak and van der Waals-type, we use the gas-phase calculations and no major change in the electronic structure is expected for molecules upon adsorption on the surface.

To understand the low threshold voltage for switching and the possible scenario of charging of molecules, we have analyzed the density of states (DOS) of positively charged and negatively charged FNAAP. The HOMO–LUMO gap of all isomers in single negative/positive charge is substantially reduced (see Supporting Information File 1, section 9 and section 10 for details of calculated DOS). The calculated HOMO–LUMO gap of charged FNAAP is smaller than that of the neutral FNAAP, which means the frontier molecular orbitals are closer to the Fermi level. The significantly smaller HOMO–LUMO gap observed for the charged species compared to their neutral counterparts suggests enhanced reactivity and a low charge injection barrier. In the case of negatively charged FNAAP, the LUMO shifts toward the Fermi level, facilitating low electron injection. In the case of positively charged molecules, the HOMO shifts toward the Fermi level and allows hole injection. These observations strongly point to the fact that molecules are in a charged state at the tunneling junction when bias is applied and require a lower threshold voltage for electron/hole injection. It is reported that if the molecules are negatively or positively charged, then the electron injection and hole injection can occur at much lower threshold voltage than that required for a neutral molecule [38–39]. As the switching is induced through the attachment of an electron or hole into the molecule, the process is referred to as electron or hole-induced switching. Depending on the polarity of the bias, either an electron is injected (at positive sample bias) or a hole (at negative sample bias). This may be directly compared to the electron or hole-catalyzed isomerization of molecules in solution [40–42].

Generally, the *E*→*Z* isomerization is fully limited without external stimulus due to the high energy barrier and endothermicity associated with the isomerization. It was shown earlier that when a positive or negative charge is attached to the molecule, the resultant energy landscape is rather shallow compared to a neutral molecule. This has been established both in solution [40–42] and on the surface [33]. That is, the isomerization is energetically less demanding once the molecule is charged. The change in the barrier is due to the drastic change in the geometry of the charged molecule. The optimized geometry of the charged FNAAP shows a drastic difference in the geometry, especially around the azo group, compared to the neutral case. The optimized geometry of the *EEE* and *ZZZ* isomers of FNAAP^+^ and FNAAP^−^, along with the neutral one, is provided in Supporting Information File 1, section 11. That is, upon charging, the potential energy landscape of isomerization changes, which supports the isomerization. As graphite–molecule interaction is weak and makes a capacitive interface between them, the molecules are likely stabilized in their ionic forms when a bias is applied at the tunneling junction. Charging of molecules on graphite [38–39,43] and ultra-thin insulating surfaces [44] is known due to the weak electronic coupling between the molecule and the graphite/insulator surface. We propose that when a sample voltage is applied to the FNAAP adlayer on graphite, they are instantaneously negatively/positively charged (depending on the voltage) due to the capacitive interface between the molecule and graphite [38–39]. The charged state of the molecule requires a lower threshold voltage for electron/hole-induced switching [38]. This scenario does not occur at molecules adsorbed on metal surfaces due to strong electronic coupling between molecules and the metal. We are unable to predict the exact number of charges in the molecule while switching, but what we could demonstrate is that charging can lead to a reduction in the HOMO–LUMO gap, as observed in the experiment. Thus, we propose that the reduction in the energy barrier and the low charge injection barrier for the charged FNAAP drives the isomerization.

For both positive and negative voltages, the switching probability increases exponentially as the voltage increases. The switching probability is directly proportional to the current flowing at the tunneling junction. As the current increases exponentially with applied voltage, it is expected that the switching probability also increases exponentially [38]. We also note that the switching probability at positive voltage is several folds larger than that at the negative voltage. This suggests that the electron injection yields a higher switching probability than that through hole injection. This could be due to high electron transport within the molecular layer compared to hole transport. We also note that the overall switching probability of FNAAP molecules in its *EEE* adlayer is several-fold higher than that for the adlayer of molecules with a single switching unit. We compared the switching probability of FNAAP with that of PABA and PyABA in the adlayer of their *E* isomer [33]. The comparison shows that the switching probability of FNAAP is an order of magnitude higher than that of PABA and PyABA. The switching experiments of PABA and PyABA were performed under similar experimental conditions. That is, by increasing the number of switching units, the overall probability of switching can be increased for molecular switches in their adlayers on surfaces. The number of switching states for FNAAP is higher (8; *EEE*, *EEZ*, *EZE*, *ZEE*, *EZZ*, *ZEZ*, *ZZE,* and *ZZZ*) compared to molecules with single switching units (2; *E* and *Z*) and thus the observed higher probability is expected. Though the switching is induced by placing the STM tip in a random position on the molecular island, each switching event observed in the time-traces corresponds to the switching/isomerization of a single molecule. That is, the calculated switching probability is per molecule. If harnessed, the increased probability of switching could be used in storing high-density of energy in the molecular system using an atomically precise write and read tool like an STM tip.

An obvious question at this stage is whether one can observe all possible 8 states and the type of interconversion between different switching states. If the tip is placed at the center, only 4 states are distinguishable: *EEE*, *EEZ*, *EZZ*, and *ZZZ*. However, all 8 states (*EEE*, *EEZ*, *EZE*, *ZEE*, *EZZ*, *ZEZ*, *ZZE* and *ZZZ*) become distinguishable if the tip is placed off-center. This is a more likely scenario due to the following reasons: we select a random position on the molecular islands for *I*–*V* measurements; due to thermal drift, the tip may move to a random location during the *I*–*V* measurements. To understand the possible switching events and the number of states distinguishable during the switching experiments, we have analyzed current versus time (time traces) at different voltage intervals. Figure 5a depicts typical time traces obtained at selected sample voltages. The time traces are obtained from *I*–*V* curves, segments of ≈100 mV and analyzed the possible number of distinguishable states. The black dashed arrowhead indicates the mean voltage corresponding to the time trace shown in the figure. Interestingly, distinguishable states (marked by magenta dashed lines) are observed at both sample voltage polarities. The statistics of the number of states as a function of sample voltage are given in Figure 5b. The number of states increases as the voltage increases and reaches a maximum value of 8. We attribute the 8 distinguishable levels of current to the 8 states of the molecule. Except for a short voltage range (−0.5 to −0.75 V and 0.6 to 0.9 V), the maximum number of switching states is accessible at all voltages. That is, the onset voltages for switching are ≈−0.5 and ≈0.6 V, which correspond to the onsets shown in Figure 4b. At this stage, we are unable to predict which current corresponds to which state; however, the clear distinguishability of states is remarkable for the experiments conducted in ambient conditions.

The average half-life of distinguishable states ranges up to a few tens of milliseconds as per the time traces (cf. Figure 5a). While metastable isomers often exhibit long lifetimes in solution [20–21] or solid-state environments [29–30], the ultra-short lifetimes of different states on the surface are surprising. During the STM experiments, electrons or holes under applied bias are continuously injected into the molecules, which vigorously drives the molecule between different possible states. That is, the half-life referred to the time-trace measurements corresponds to a constant external trigger, at a given bias and a given current. If the bias (and thus the current) is removed, it is plausible that the molecule could reside in a specific state for a longer duration, closer to its intrinsic thermal stability. We have observed that photo- and thermal-triggered AB and azopyrazole derivatives remain in the *Z*-isomeric state for several hours on the surface [45]. These exceptional lifetimes of non-equilibrium states of FNAAP are also particular for the condensed state on the surface, due to the confinement of several degrees of freedom when condensed on the surface. It is also noted that the change in current for switching between states is higher (≈ one order of magnitude larger) for the positive sample voltage compared to negative sample voltage. This is also obvious in the cumulative number of states at positive sample bias compared to negative sample bias. This suggests that different states are better distinguished at positive sample voltage.

The following is understandable about the switching pattern from the time traces. (a) A stepwise change in current is observed generally, indicating that the molecule can switch between any given state, or any order does not exist for the switching. (b) The molecule can be switched from the lowest current state to the highest current state (cf. time trace taken at 1.1 V) without any intermediates. (c) All 8 states are accessible reversibly even at lower sample voltages, indicating that switching can be performed between any given state at a low threshold voltage. That is, all states are accessible and are remarkably stable at all sample voltages. It is concluded that the high energy barrier and the long lifetime of the non-equilibrium states are at the origin of their remarkable stability at room temperature. The remarkable stability of the states at ambient conditions provides an opportunity to use a single FNAAP molecule as an 8-bit operation. We propose to switch the molecule using an STM tip, which is a tool that can manipulate molecules at an atomic level. Thus, the selection of a single molecule and the selection of a part of molecules is not impractical. Once the selection is off-centered all isomers are distinguishable and non-degenerate. The molecule may be switched using an STM tip between multiple states and the state could be read using the magnitude of the current. Given the dimension of the molecule (≈2 nm^2^ as obtained from the unit cell) one could accommodate ≈200 Tbits in a 1 cm^2^ area. Such a remarkable density of bits is possible since each molecule acts as an 8-bit operation unit. If the states of the molecule are addressed as |1⟩, |2⟩,…,|8⟩, one can use combinations of different states of molecules in an array to record information. For example, if FNAAP is in states |2⟩, the operation is addressed as 0 1 0 0 0 0 0 0, which could be assigned to a number, letter, etc. Depending on the state of the adjacent molecules in an array, one can assign a combination of states to address number, letters etc. Thus, in an array of 8 molecules, 8^8^ permutations are possible.

## Conclusion

We have investigated the microscopic structure and electron/hole-induced conformational switching of a tripodal azopyrazole derivative (FNAAP) in its ultra-thin films on a HOPG surface at ambient conditions. Most of the molecules (>98%) in the film are in the *EEE* configuration and form a hexagonal close-packed assembly. Molecules within the condensed phase of *EEE* isomeric states were switched between different isomers reversibly using electrons/holes injected from an STM tip. The electron/hole-induced switching is characterized by the observation of a threshold voltage for switching. The observed threshold voltages are lower than expected and indicate that the switching is mediated through a charged state of FNAAP. The observed switching probability is several-fold higher than that of other AB derivatives with one switching unit. The analysis of the number of switchable states as a function of sample voltage reveals 8 maximum states. These states have distinguishable current values and are assigned to *EEE*, *EEZ*, *EZE*, *ZEE*, *EZZ*, *ZEZ*, *ZZE,* and *ZZZ*. The clear distinguishability of these states is remarkable for the experiments conducted in ambient conditions. The change in current for switching between states is higher (≈ one order of magnitude larger) for positive sample voltage compared to negative sample voltage. The cumulative switching per unit voltage is higher at positive sample bias compared to negative sample bias. The remarkable stability of the states at ambient conditions provides an opportunity to use a single FNAAP molecule as an 8-bit operation and one could accommodate ≈200 Tbits in a 1 cm^2^ area.

## Experimental

### Preparation of adlayer

The FNAAP molecule is synthesized as per our previous report [29]. Ultra-thin films were prepared by drop casting from ethanolic solutions of molecules (concentration between 10^−5^ to 10^−6^ M) on freshly cleaved highly oriented pyrolytic graphite (HOPG, ZYB grade from μMasch). Approximately 4 μL of solutions of FNAAP were drop-casted on the HOPG surface, dried (a few minutes) in ambient conditions, and AFM/STM studies were performed. Relative humidity (≈50%) and temperature (22–25 °C) of the room were controlled by an air conditioner and a dehumidifier. While drop casting, the sample was kept at ≈20–30 °C to ensure a smooth flow of the solvent over the substrate. After drop casting, all films were subjected to vacuum pumping (typically 0.01 mbar) for about 1 h. Before depositing molecules, freshly cleaved graphite surfaces were subjected to control AFM experiments to check for any contamination or impurities on the surface. We also note that no traces of ethanol remained on the surface after pumping, as confirmed by AFM images.

### Instrumentation

All AFM and STM measurements were performed using an Agilent 5500 in soft tapping mode and an ATM300 from RHK Technology, respectively. Aluminum-coated silicon cantilevers from Nanosensors (PPP-NCHR) were used as AFM probes. The force constant and resonance frequency of the cantilevers during the imaging were 30–34 N/m and ≈300 kHz, respectively. A Pt/Ir (80:20) tip was mechanically prepared and used as a probe for STM imaging. The XY and Z scales of STM images are calibrated using the graphite lattice. All the AFM and STM images are post-processed using WSxM from Nanotec [46]. Mesh averaging implemented in WSxM [46] is used for enhancing the contrast of STM images. Current–voltage (*I*–*V*) measurements and statistics of switching are performed in ambient conditions. The voltage is referred to with respect to the sample. Similar set point voltage and current were chosen for the *I*–*V* measurements included in the statistics in order to ensure that the relative distance between the tip and the molecules is similar while performing the *I*–*V* measurements. Due to thermal drift during the *I*–*V* measurement, it is assumed that each *I*–*V* measurement includes the switching of a few molecules under the tip. However, every switching event corresponds to isomerization in a single molecule. To overcome the effect of drift, *I*–*V* measurements are recorded within an average time of ≈4 s. This ensures that the location of the *I*–*V* measurement does not drastically drift from the originally selected point.

### Details of force field simulation and computations

The Reax Force Field method implemented in Quantumwise ATK-classical is devised for geometry optimization. The graphite (previously optimized) was kept fixed during the simulations, and the molecular geometry was fully optimized. Parameter set: ReaxFF-CHO-2008 [32]. A force tolerance of 0.001 eV/Å, stress tolerance of 0.001 eV/Å, and a maximum step size 0.01 Å were used for the optimization. The limited memory BFGS (L-BFGS) optimizer method was employed for optimization. L-BFGS is an optimization algorithm in the family of quasi-Newton that approximates the Broyden–Fletcher–Goldfrab–Shanno (BFGS) algorithm using a limited amount of computer memory. A pre-optimized two-layer graphite was used from the library data as provided in the Quantumwise DFT package. The lattice parameters for graphite are, *a* = 2.4612 Å, *b* = 2.4612 Å, *c* = 6.709 Å, and α = 60°. Molecular orbitals were obtained from the calculations performed using the Gaussian 03 program suite [47].

## Supporting Information

AFM topographs of ultra-thin film of FNAAP, height profile of FNAAP, AFM phase images of ultra-thin films of FNAAP, UV–vis spectrum of FNAAP, STM image of ultra-thin films of FNAAP, model for the incommensurate hexagonal adlayer of FNAAP on graphite, *I*–*V* measurements performed on adlayer of FNAAP, HOMO–LUMO gap of different isomers of FNAAP, DOS of *EEE* and *EEZ* isomers of FNAAP, DOS of *EZZ* and *ZZZ* isomers of FNAAP. Optimized charged geometries of *EEE*- and *ZZZ*-isomers of FNAAP. Chemical structures of *EEE*- and *ZZZ*-isomers of FNAAP and the azo derivatives used for comparison.

File 1 Additional information and spectra.

## Data Availability

All data that supports the findings of this study is available in the published article and/or the supporting information of this article.
