# Supplementary material for: Highly distinguishable isomeric states of a tripodal arylazopyrazole derivative on graphite through electron/hole-induced switching at ambient conditions
Source: Beilstein J Org Chem. 2025 Jul 22;21:1496–507. doi: 10.3762/bjoc.21.112 (PMC12302408; doi:10.3762/bjoc.21.112)
Supplement: File 1 — Additional information and spectra. [file Beilstein_J_Org_Chem-21-1496-s001.pdf]

BEILSTEIN JOURNAL OF ORGANIC CHEMISTRY

Supporting Information

for

Highly distinguishable isomeric states of a tripodal arylazopyrazole derivative on graphite through electron/hole-induced switching at ambient conditions

Himani Malik, Sudha Devi, Debapriya Gupta, Ankit Kumar Gaur, Sugumar Venkataramani and Thiruvancheril G. Gopakumar

Beilstein J. Org. Chem. **2025,** *21,* 1496–1507. doi:10.3762/bjoc.21.112

Additional information and spectra

License and Terms: This is a supporting information file under the terms of the Creative Commons Attribution License (https://creativecommons.org/licenses/by/4.0). Please note that the reuse, redistribution and reproduction in particular requires that the author(s) and source are credited and that individual graphics may be subject to special legal provisions.
The license is subject to the *Beilstein Journal of Organic Chemistry* terms and conditions: (https://www.beilstein-journals.org/bjoc/terms)

Table of contents

S No	Content	Page number
S1	AFM topographs of ultra-thin film of FNAAP figure	S2
S2	Height profile of molecular islands of FNAAP	S2
S3	Additional AFM phase images of ultra-thin films of FNAAP	S3
S4	UV–vis spectrum of FNAAP	S4
S5	STM image of ultra-thin films of FNAAP	S5
S6	Model for the incommensurate hexagonal adlayer of FNAAP on graphite	S6
S7	I–V measurements performed on adlayer of FNAAP	S7
S8	HOMO–LUMO gap of different isomers of FNAAP	S8
S9	DOS of *EEE* and *EEZ* isomers of FNAAP	S9
S10	DOS of *EZZ* and *ZZZ* isomers of FNAAP	S10
S11	Optimized geometries of neutral and charged *EEE*- and *ZZZ*-isomers of FNAAP	S11
S12	Chemical structures of *EEE*- and *ZZZ*-isomers of FNAAP and the azo derivatives used for comparison	S12

S1: AFM topographs of ultra-thin film of FNAAP figure

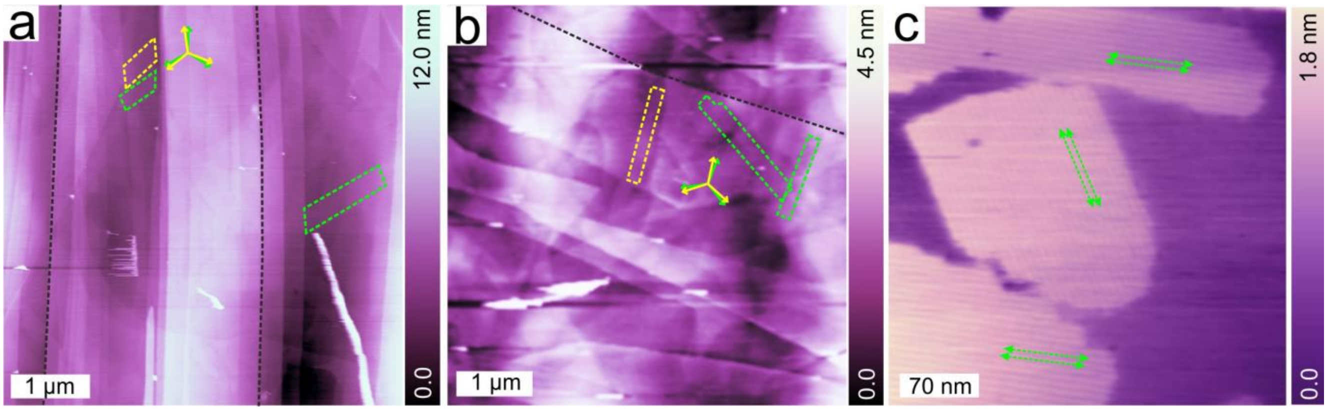

Figure S1: (a–c) AFM topographs of ultra-thin film of FNAAP on HOPG (0001) surface deposited from ethanol at ambient conditions. The topographs correspond to the phase images provided in Figure 2a, b and c in the main article. Molecular islands are marked with dashed green and dashed yellow lines. Black dashed lines indicate a few terrace edges. (c) High-resolution AFM topograph of FNAAP. Line-like contrast corresponding to molecular super-lattice is marked by green double headed arrows.

S2: Height profile of molecular islands of FNAAP

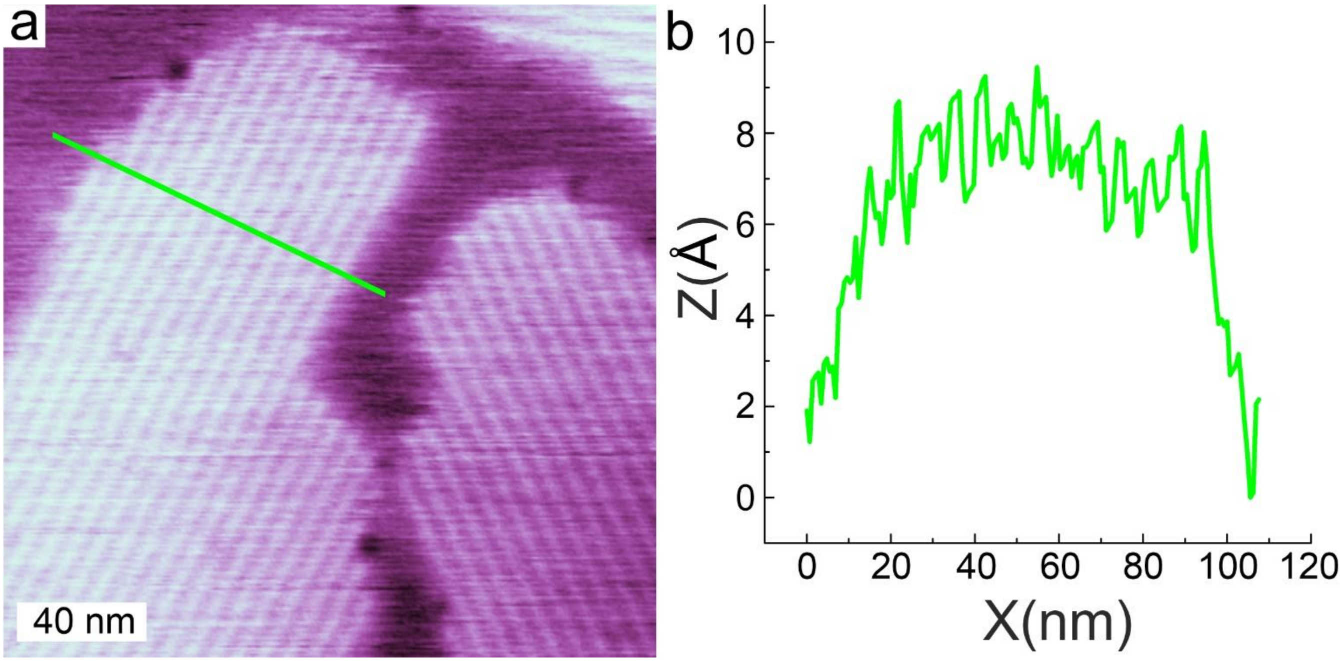

Figure S2: (a) AFM topograph of monolayer film of FNAAP. (b) The height profile taken along the indicated lines. The apparent height of the molecular islands is ≈0.7 nm and is typical for monolayer film of a planar molecule.

S3: Additional AFM phase images of ultra-thin films of FNAAP

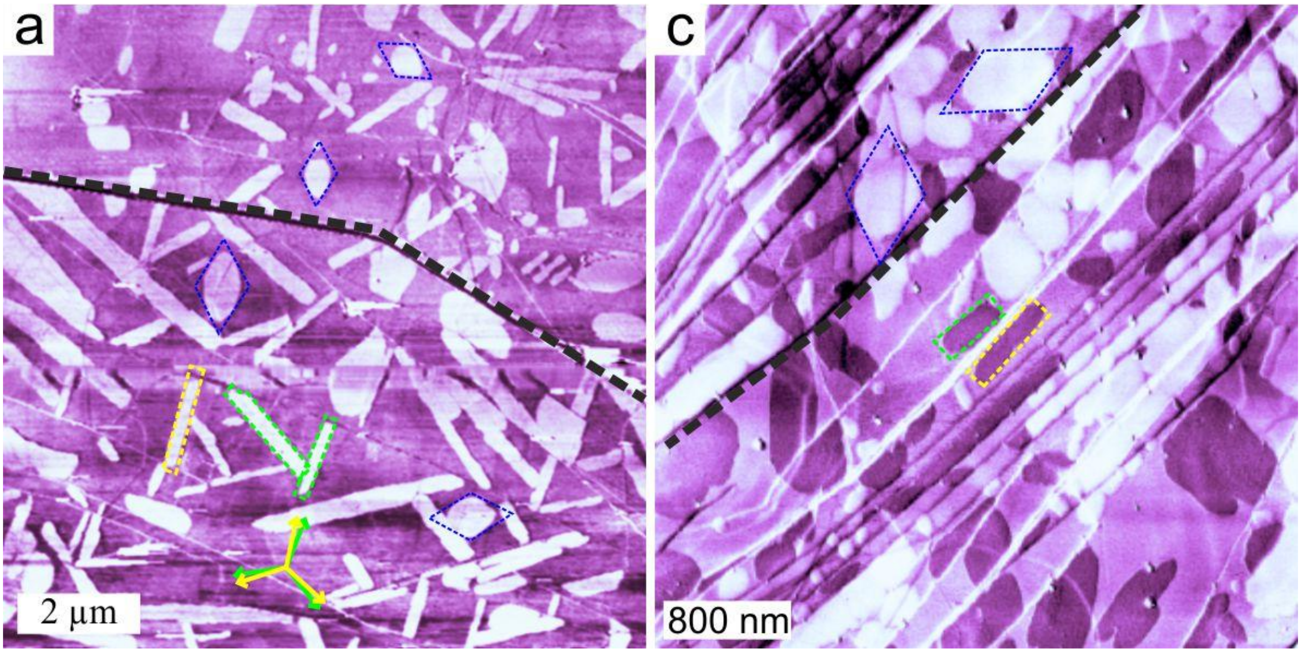

Figure S3: (a, b) AFM phase images of ultra-thin film of FNAAP on HOPG (0001) deposited from ethanol at ambient conditions. Molecular islands are marked with green dashed lines. Black dashed lines indicate a few terrace edges.

S4: UV–vis spectrum of FNAAP

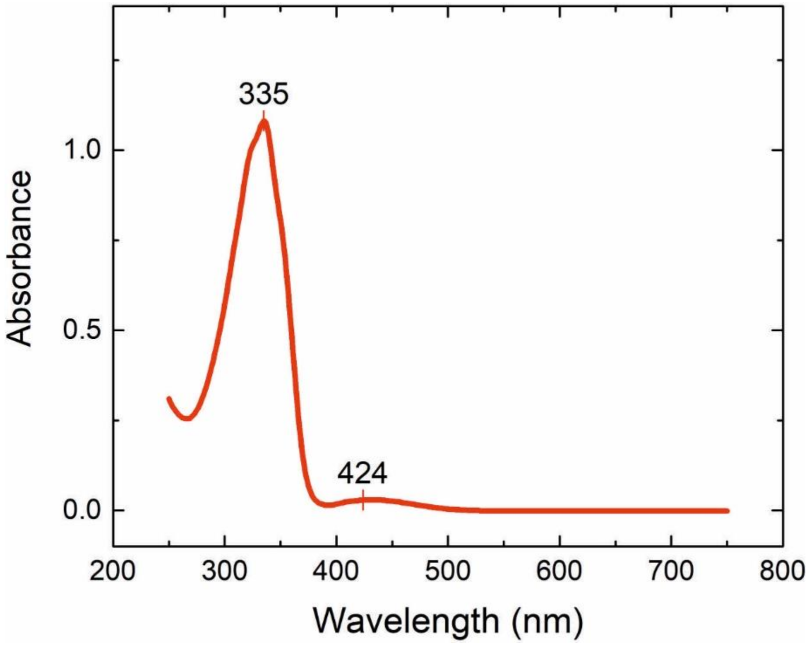

Figure S4: UV–vis absorbance spectrum of FNAAP in ethanol/chloroform 1:1 mixture. 335 nm and 424 nm indicate the major resonances corresponding to π–π* and n–π* absorptions of the *EEE*-isomer.

S5: STM image of ultra-thin films of FNAAP

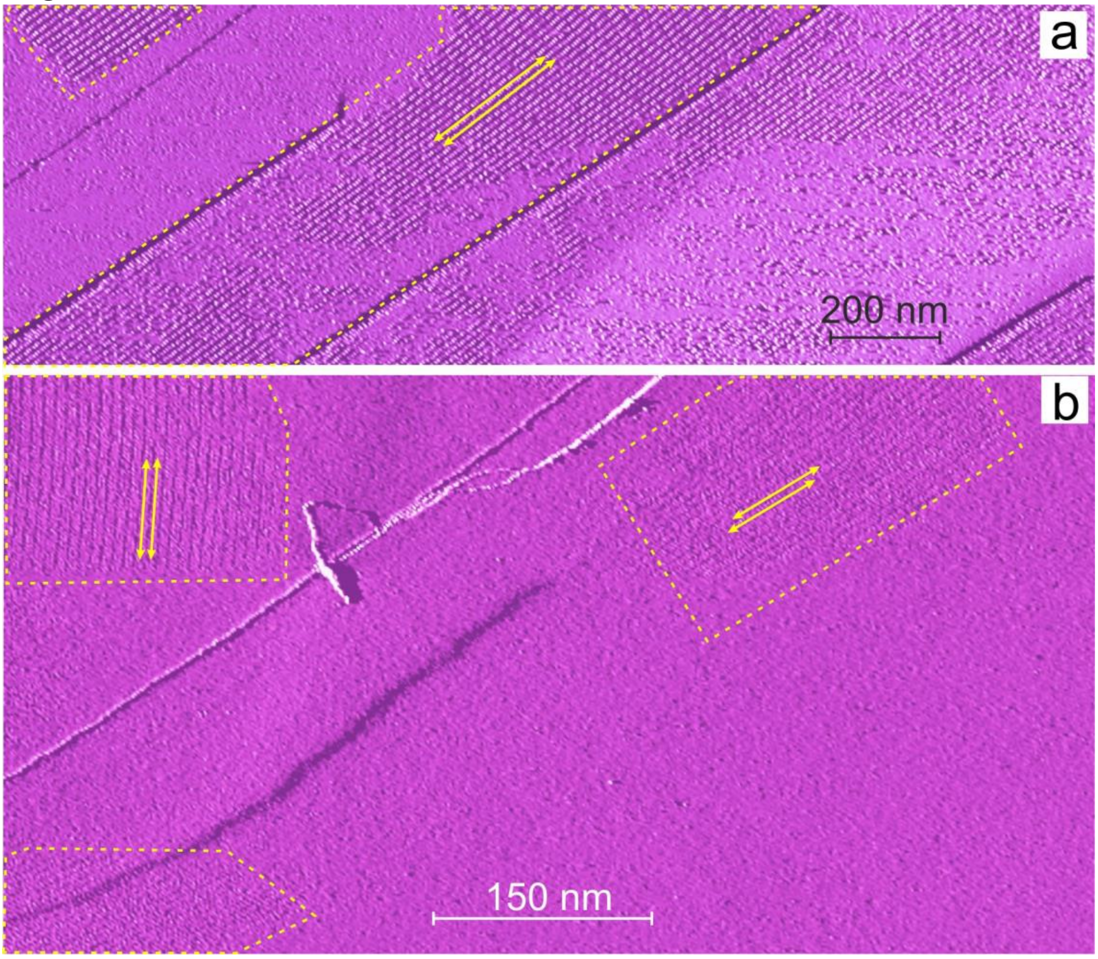

Figure S5: (a, b) STM current images (300 pA, 0.65 V) of ultra-thin film of FNAAP on HOPG (0001) deposited from ethanol at ambient conditions. The images are acquired from two independent areas. Yellow dashed lines depict molecular islands. The typical shape of the islands resembled the 1D islands as observed in AFM. Since the majority of the surface is covered by 1D phase (≈88% as deduced using AFM images), it is likely assumed that the islands are originating from 1D phase. Super-periodic pattern can be visualized within the islands and are marked by yellow double headed lines. The distance between the super-periodic pattern is ≈6.1 nm and is well in agreement with the AFM measurements and models. The angle between the long edge of the islands (≈0°, ≈120°) are also consistent to the observation made from AFM.

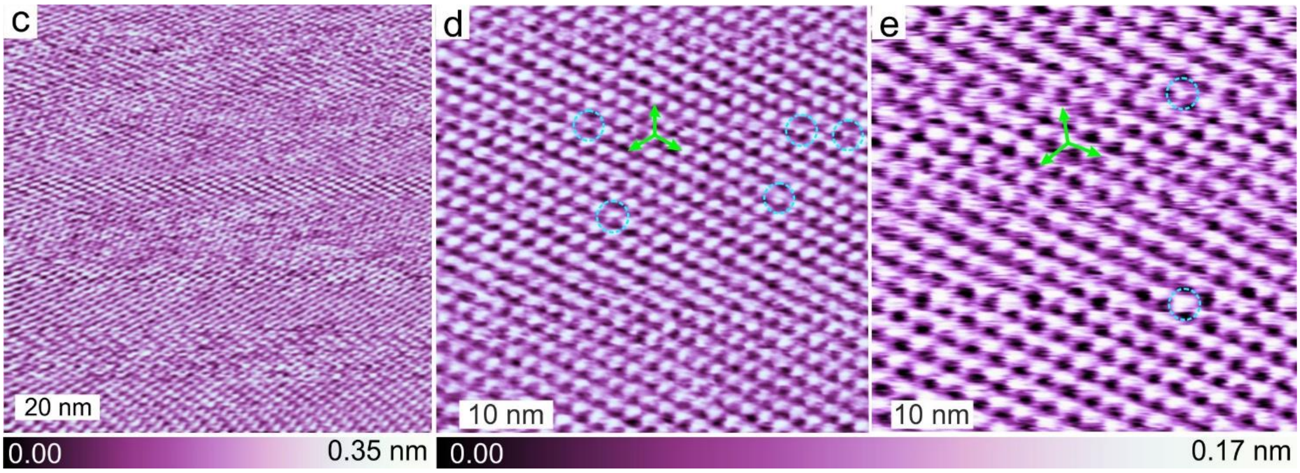

Figure S6: (c) STM topography (300 pA, 0.3 V) of ultra-thin film of FNAAP on HOPG (0001) deposited from ethanol at ambient conditions. (d, e) High-resolution STM images. Threefold green arrowheads show the molecular lattice directions. Typically, the molecular contrast appears as a bright symmetric spot. We observed the molecular feature with distinct appearance at certain locations. A few of such locations are marked by cyan circles. We presume that such contrast difference in molecules is due to different conformations of the molecules.

S6: Model for the incommensurate hexagonal adlayer of FNAAP on graphite.

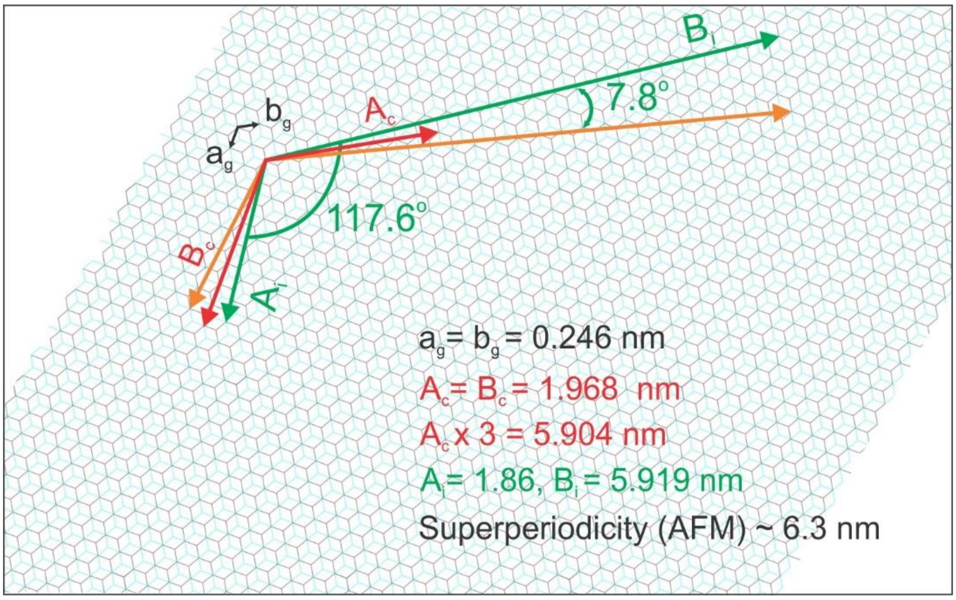

Figure S7: Geometrical model for the incommensurate and commensurate hexagonal adlayer of FNAAP on graphite. Graphite lattice is shown using hexagonal wire mesh with grey and cyan representing the adjacent layers. a_g and b_g are lattice vectors of graphite and the angle between these vectors is 120°. A and B represent the lattice vectors of the molecular adlayer. The superlattice vector (A_i/B_i) formed by an incommensurate lattice of molecule with respect to graphite is depicted using yellow and green arrows. A_i = 1.86 nm and B_i = 5.904 nm as deducted using a reference commensurate lattice. The commensurate lattice is indicated using red arrows. These lattice parameters of the incommensurate lattice correspond to the periodicity of the moiré pattern. Since the incommensurate lattice has one long periodicity (5.904 nm) and one short periodicity (1.86 nm), the AFM resolves only the long periodicity and thus the moiré pattern of the 1D islands appear as long like-like features. The angle between the incommensurate lattice vectors is 117.6°. As revealed by STM, the molecular adlayer has a nearly hexagonal symmetry. One of the incommensurate lattices is making an angle of 3.9° with respect to graphite lattice and is matching with the experimental observation. Mirror domains are rotated by ≈3.5° with respect to the graphite lattice as deduced from the AFM images. The commensurate lattice (A_c = B_c = 1.968 nm) does not lead to any moiré pattern as it forms no super-lattice. We propose that the 2D islands are originating from a commensurate lattice and are not showing any moiré pattern.

S7: I–V measurements performed on adlayer of FNAAP

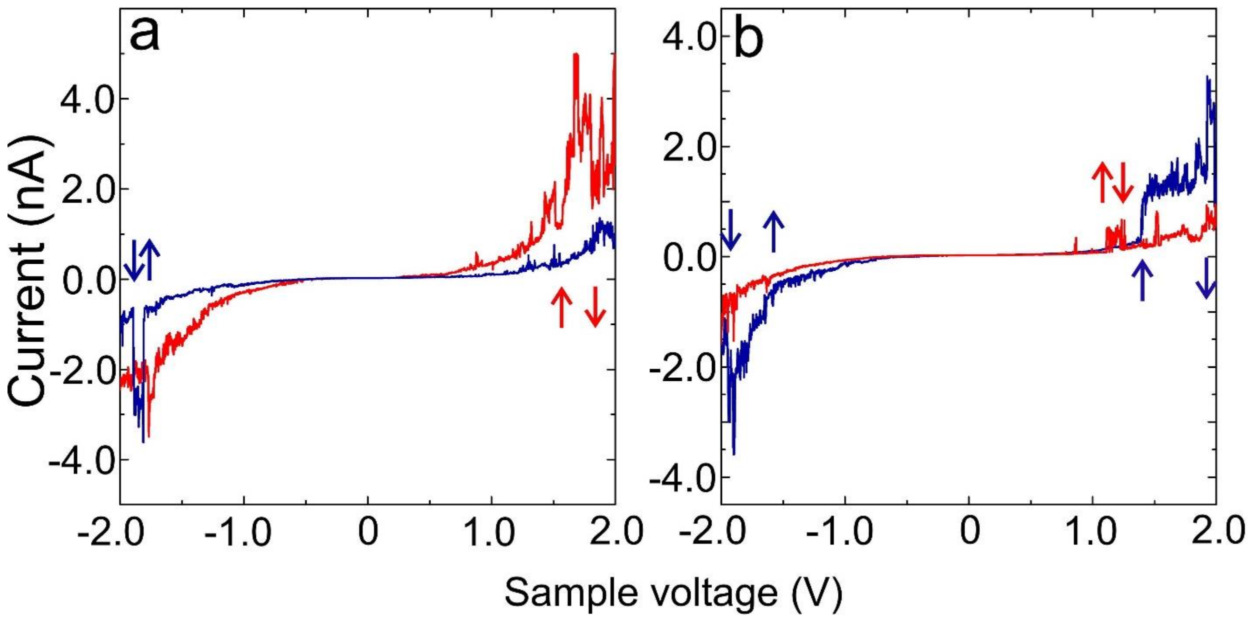

Figure S8: Current–voltage (I–V) measurements (a, b) performed on adlayer of FNAAP. Red and blue lines represent two independent I–V curves. Switching of the molecules is indicated by red and blue arrows (up and down events).

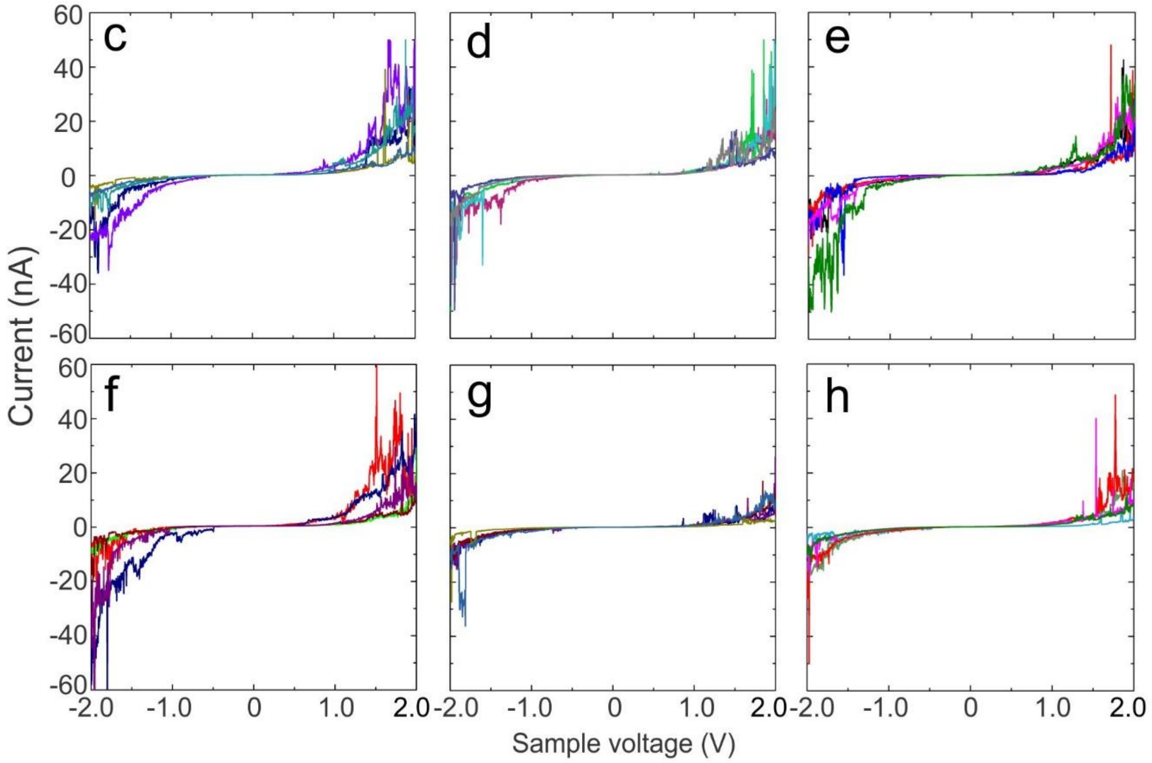

Figure S9: Current–voltage (I–V) measurements (c, d, e, f, g, h) performed on adlayer of FNAAP. Different colours in each set (5 curves) represent independent I–V curves recorded on molecular islands. We have used around 160 selected I–V curves for the statistical analysis.

S8: HOMO–LUMO gap of different isomers of FNAAP

**Molecules**	**HOMO–LUMO gap (in eV)**		
	**$FNAAP^{+}$**	**FNAAP**	**$FNAAP^{-}$**
EEE	2.85	3.60	3.06
EEZ	1.50	3.27	2.86
EZZ	2.07	3.26	2.77
ZZZ	2.52	3.35	2.63

Table S1: HOMO–LUMO gap of isomers of FNAAP in charged (negative/positive) states. All calculations are performed at the B3LYP/6-311g level of theory using Gaussian 09.

S9: DOS of *EEE* and *EEZ* isomers of FNAAP

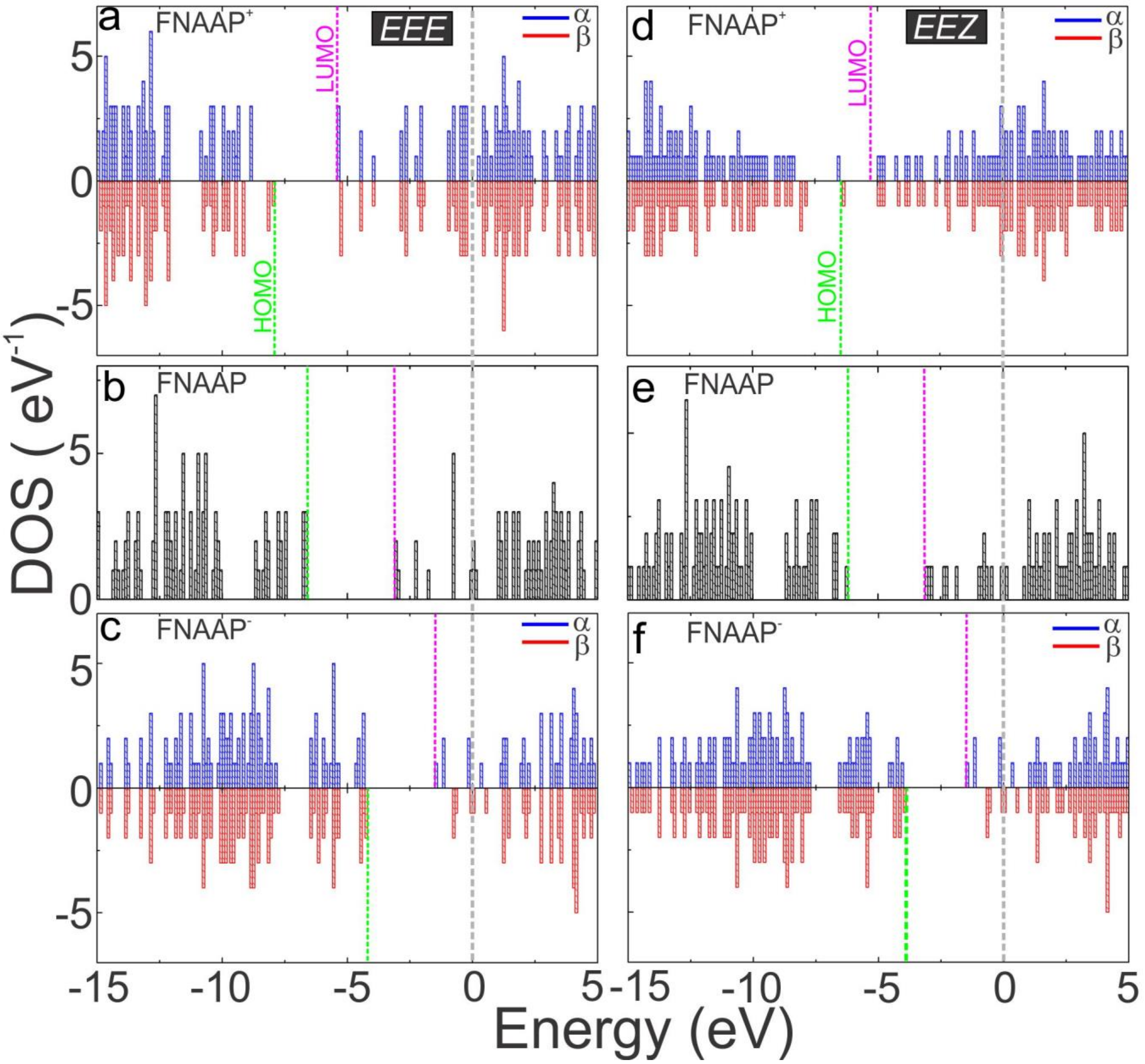

Figure S10: Density of states (DOS) of the positive, neutral, and negative state of the *EEE*-isomer (a,b,c) and *EEZ*-isomer (d,e,f) of FNAAP. The DOS is plotted per 0.1 eV. The spin states of the charged cases are depicted using (blue) and (red). HOMO and LUMO are marked by green and magenta dashed lines, respectively. Light grey line represents vacuum energy.

S10: DOS of *EZZ* and *ZZZ* isomer of FNAAP

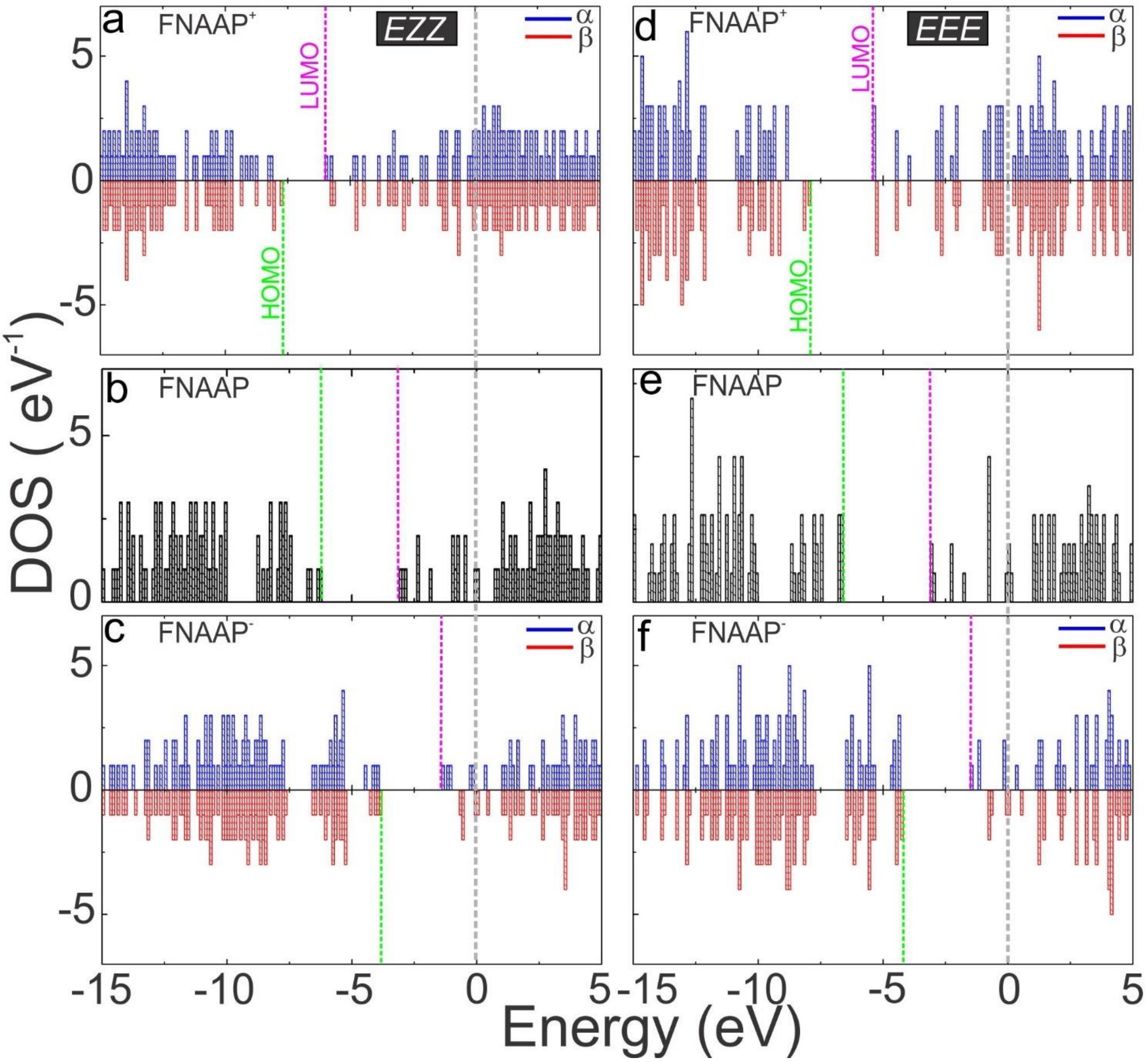

Figure S11: Density of states (DOS) of the positive, neutral, and negative states of the *EZZ*-isomer (a,b,c) and *ZZZ*-isomer (d,e,f) of FNAAP. The DOS is plotted per 0.1 eV. The spin states of the charged cases are depicted using (blue) and (red). HOMO and LUMO are marked by green and magenta dashed lines, respectively. Light grey line represents vacuum energy.

S11: Optimized geometries of neutral and charged *EEE*- and *ZZZ*-isomers of FNAAP

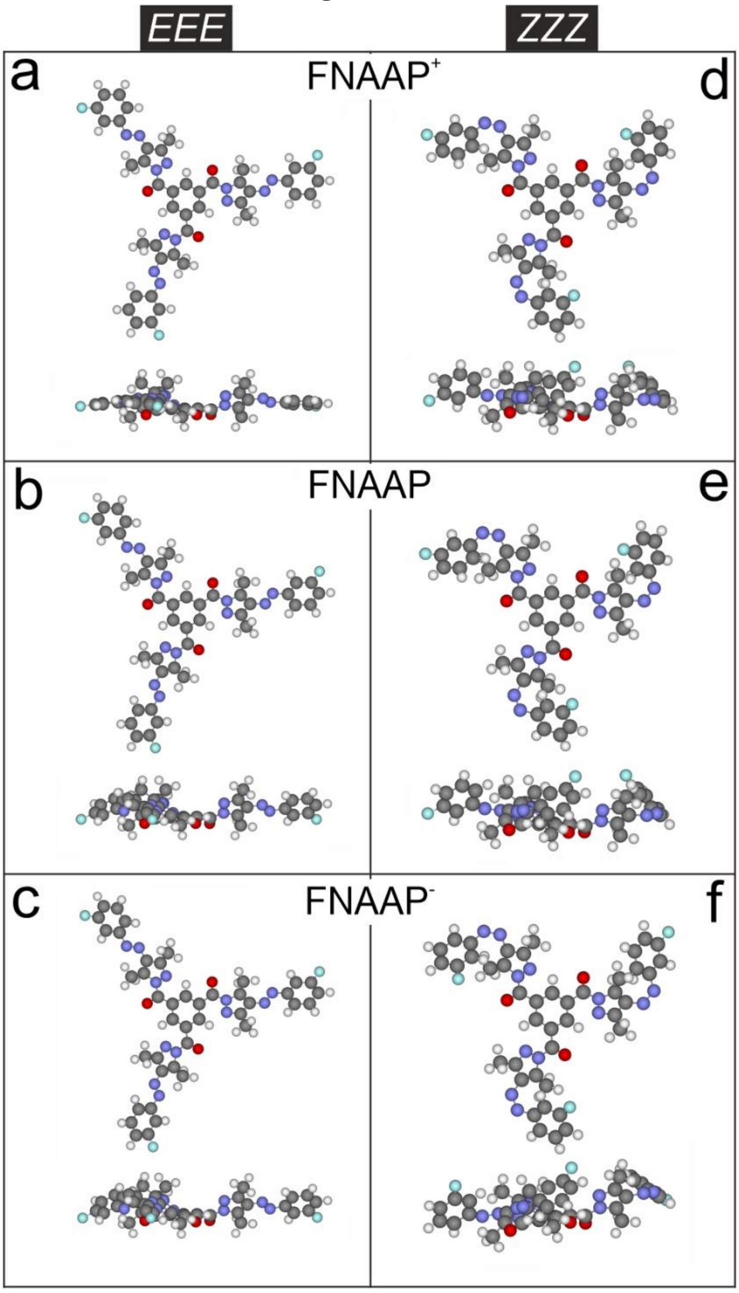

Figure S12: Geometry-optimized ball and stick models of the positive, neutral, and negative state of the *EEE*-isomer (a,b,c) and *ZZZ*-isomer (d,e,f) of FNAAP are presented with top and side view.

S12: Chemical structures of *EEE*- and *ZZZ*- isomers of FNAAP and the azo derivatives used for comparison.

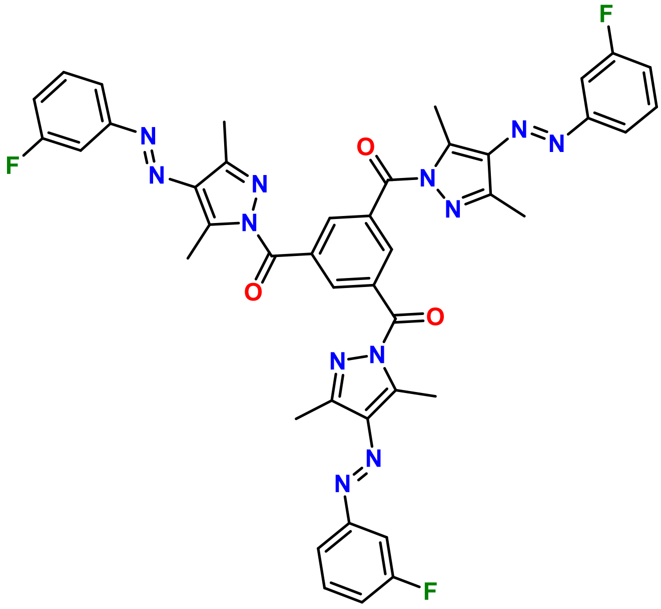

***EEE*-FNAAP**

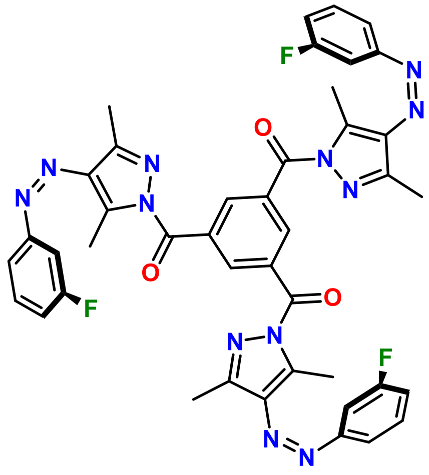

***ZZZ*-FNAAP**

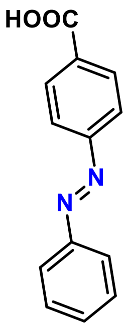

PABA

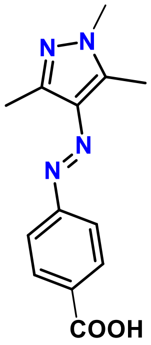

PyABA

Figure S13: Chemical structures of *EEE*- and *ZZZ*-isomers of FNAAP and the azo derivatives used for comparison.
